# *N*-(2,4,6-Trimethyl­phen­yl)formamide

**DOI:** 10.1107/S1600536810051469

**Published:** 2010-12-11

**Authors:** Marilé Landman, Belinda van der Westhuizen, Daniela I. Bezuidenhout, David C. Liles

**Affiliations:** a Department of Chemistry, University of Pretoria, Private Bag X20, Hatfield 0028, South Africa

## Abstract

The title compound, C_10_H_13_NO, was obtained as the unexpected, almost exclusive, product in the attempted synthesis of a manganese(I)–*N*-heterocyclic carbene (NHC) complex. The dihedral angle between the planes of the formamide moiety and the aryl ring is 68.06 (10)°. In the crystal, mol­ecules are linked by N—H⋯O hydrogen bonds, forming infinite chains along the *c* axis.

## Related literature

For background to formamide formation from NHCs, see: Denk *et al.* (2001 ▶). The rotation of the formamide entity out of the plane of the aryl ring and the hydrogen-bonding motif displayed by this structure are similar to those observed for the related compound *N*-(2,6-dimeth­yl)-formamide, see: Hanson *et al.* (2004 ▶); Omondi *et al.* (2005 ▶).
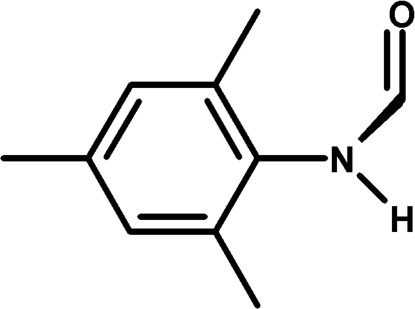


## Experimental

### 

#### Crystal data

C_10_H_13_NO
*M* *_r_* = 163.21
Monoclinic, 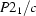

*a* = 8.0659 (7) Å
*b* = 15.9004 (13) Å
*c* = 8.4290 (7) Å
β = 119.361 (1)°
*V* = 942.17 (14) Å^3^
*Z* = 4
Mo *K*α radiation
μ = 0.07 mm^−1^
*T* = 293 K
0.44 × 0.38 × 0.28 mm

#### Data collection

Bruker (Siemens) P4 diffractometer fitted with a SMART 1K CCD detector
Absorption correction: multi-scan (*SADABS*; Bruker, 2001 ▶) *T*_min_ = 0.946, *T*_max_ = 0.979
4988 measured reflections
1778 independent reflections
1607 reflections with *I* > 2σ(*I*)
*R*_int_ = 0.026

#### Refinement

*R*[*F* ^2^ > 2σ(*F* ^2^)] = 0.048
*wR*(*F* ^2^) = 0.138
*S* = 1.09
1778 reflections
161 parameters
All H-atom parameters refined
Δρ_max_ = 0.21 e Å^−3^
Δρ_min_ = −0.21 e Å^−3^

### 

Data collection: *SMART* (Bruker, 2001 ▶); cell refinement: *SAINT* (Bruker, 2001 ▶); data reduction: *SAINT*; program(s) used to solve structure: *SHELXTL* (Sheldrick, 2008 ▶); program(s) used to refine structure: *SHELXTL* and *SHELXL97* (Sheldrick, 2008 ▶); molecular graphics: *POV-RAY* (Cason, 2004 ▶) and *Mercury* (Bruno *et al.*, 2002 ▶); software used to prepare material for publication: *SHELXL97* and *PLATON* (Spek, 2009 ▶).

## Supplementary Material

Crystal structure: contains datablocks I, global. DOI: 10.1107/S1600536810051469/bt5433sup1.cif

Structure factors: contains datablocks I. DOI: 10.1107/S1600536810051469/bt5433Isup2.hkl

Additional supplementary materials:  crystallographic information; 3D view; checkCIF report

## Figures and Tables

**Table 1:** Hydrogen-bond geometry (Å, °)

*D*—H⋯*A*	*D*—H	H⋯*A*	*D*⋯*A*	*D*—H⋯*A*
N1—H1⋯O1^i^	0.83 (2)	2.05 (2)	2.8775 (18)	171.4 (19)

Symmetry code: (i) 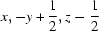.

**Fig. 1.**
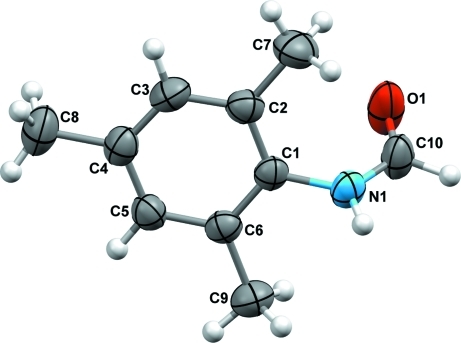
The molecular structure of 1 showing the atomic numbering scheme. Displacement ellipsoids are shown at the 50% probability level and H atoms are shown as small spheres of arbitrary radii.

**Fig. 2.**
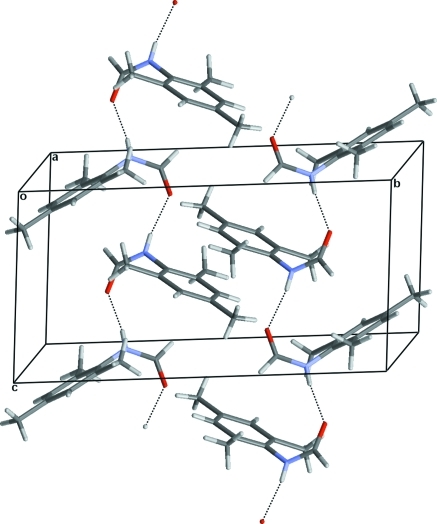
Packing diagram of 1 viewed approximately down the a-axis and showing O—H···O hydrogen bonding interactions which link molecules to form infinite chains.

## supplementary crystallographic information

### Comment

*N*-(2,4,6-Trimethyl-phenyl)-formamide (*N*-mesityl-formamide) (1) was formed as an unexpected product in the attempted synthesis of a manganese(I)—N-heterocyclic carbene (NHC) complex. Instead of the target complex, the mesityl formamide was obtained almost exclusively. The ylidene molecule, formed by deprotonation of 1,3-bis(2,4,6-trimethyl-phenyl)-imidazolium chloride (IMesHCl) by a strong base, is prone to undergo side reactions. Thus the strong base, and the subsequent addition of Mn(CO)_5_Br, resulted in the formation of *N*,*N*'-bis-mesityl-*N*-vinyl-formamidine and after hydrolysis of this molecule the NC—N bond dissociated to form 1 and a mesityl-vinyl-amine fragment which was not isolated. Denk *et al.* (2001) have reported the hydrolysis of NHCs, with formamide formation *via* ring opening, resulting in an acyclic product.

The molecular structure of the title compound (1) (Fig. 1) is similar to that of the related compound, *N*-(2,6-dimethyl-phenyl)-formamide, the structure of which has been reported at 173 K (Hanson, *et al.*, 2004) and 293 K (Omondi, *et al.*, 2005). Owing to the influence of the bulky methyl substituents in the 2 and 6 positions, the formamide moiety is rotated out of the plane of the aryl ring: in 1, the angle between the planes of the formamide moiety (C1, N1, C10, O1) and the aryl ring is 68.06 (10)°. This compares with 64.75 (12)° (173 K) and 66.45 (12)° (293 K) found for the 2,6-dimethyl analogue.

In the formamide moieties of both structures the O atom is *trans* to N—H thus allowing the molecules to be linked to form infinite chains by N—H···O hydrogen bonds. However the spatial arrangements within the chains differ. In the 2,6-dimethyl analogue (space group *P*2_1_2_1_2_1_), the axis of each chain is parallel to the *a* unit cell axis and neighbouring molecules within a chain are related by the *a*-axial unit cell translation. Thus the aryl ring of each molecule is parallel to those of its neighbours within the chain and they are stacked one above the other but with a step-wise offset. In contrast, in 1, the axis of each chain is parallel to the *c* unit cell axis and neighbouring molecules within a chain are related by a *c*-glide plane. Thus neighbouring molecules in a chain are arranged on opposite sides of the chain axis and the aryl rings are not mutually parallel (Fig. 2).

### Experimental

Mn(CO)_5_Br (3 mmol, 0.74 g) and Me_3_NO (2.8 mmol, 0.21 g) were stirred in thf resulting in a red solution. IMesHCl (3 mmol, 1.02 g) was deprotonated in thf by the addition of base (3 mmol) and the ylidene was added to the solution and stirred overnight. The thf solvent was removed and the products were separated on an aluminium oxide 90 (alox) column. Elution with dichloromethane (dcm) and thf yielded starting material and a yellow fraction respectively. The yellow fraction was crystallized from a saturated chloroform solution to give an unexpected organic product, *N*-mesityl-formamide (1, C_10_H_13_NO). ^1^H NMR (δ, p.p.m.), C_6_D_6_: 2.24 (br, 9H), 3.85 (br, 1H), 6.65 (br, 2H), 8.32 (br, 1H); ^13^C NMR (δ, p.p.m.), C_6_D_6_: 18.8, 21.2, 129.2, 130.2, 135.3, 137.6, 208.6.

### Refinement

The coordinates and individual *U_iso_* parameters for all H atoms were freely refined.

### Crystal data

**Table:**

C_10_H_13_NO	*F*(000) = 352
*M**_r_* = 163.21	*D*_x_ = 1.151 Mg m^−^^3^
Monoclinic, *P*2_1_/*c*	Mo *K*α radiation, λ = 0.71073 Å
Hall symbol: -P 2ybc	Cell parameters from 3714 reflections
*a* = 8.0659 (7) Å	θ = 2.8–26.4°
*b* = 15.9004 (13) Å	µ = 0.07 mm^−^^1^
*c* = 8.4290 (7) Å	*T* = 293 K
β = 119.361 (1)°	Prism, colourless
*V* = 942.17 (14) Å^3^	0.44 × 0.38 × 0.28 mm
*Z* = 4	

### Data collection

**Table:**

Bruker P4 diffractometer	1778 independent reflections
Radiation source: fine-focus sealed tube	1607 reflections with *I* > 2σ(*I*)
graphite	*R*_int_ = 0.026
Detector resolution: 8.3 pixels mm^-1^	θ_max_ = 26.4°, θ_min_ = 2.6°
φ and ω scans	*h* = −9→10
Absorption correction: multi-scan (*SADABS*; Bruker, 2001)	*k* = −14→18
*T*_min_ = 0.946, *T*_max_ = 0.979	*l* = −10→5
4988 measured reflections	

### Refinement

**Table:**

Refinement on *F*^2^	Primary atom site location: structure-invariant direct methods
Least-squares matrix: full	Secondary atom site location: difference Fourier map
*R*[*F*^2^ > 2σ(*F*^2^)] = 0.048	Hydrogen site location: difference Fourier map
*wR*(*F*^2^) = 0.138	All H-atom parameters refined
*S* = 1.09	*w* = 1/[σ^2^(*F*_o_^2^) + (0.0738*P*)^2^ + 0.186*P*] where *P* = (*F*_o_^2^ + 2*F*_c_^2^)/3
1778 reflections	(Δ/σ)_max_ = 0.004
161 parameters	Δρ_max_ = 0.21 e Å^−^^3^
0 restraints	Δρ_min_ = −0.21 e Å^−^^3^
0 constraints	

### Special details

**Table:**

Geometry. All e.s.d.'s (except the e.s.d. in the dihedral angle between two l.s. planes) are estimated using the full covariance matrix. The cell e.s.d.'s are taken into account individually in the estimation of e.s.d.'s in distances, angles and torsion angles; correlations between e.s.d.'s in cell parameters are only used when they are defined by crystal symmetry. An approximate (isotropic) treatment of cell e.s.d.'s is used for estimating e.s.d.'s involving l.s. planes.
Refinement. Refinement of *F*^2^ against ALL reflections. The weighted *R*-factor *wR* and goodness of fit *S* are based on *F*^2^, conventional *R*-factors *R* are based on *F*, with *F* set to zero for negative *F*^2^. The threshold expression of *F*^2^ > 2σ(*F*^2^) is used only for calculating *R*-factors(gt) *etc*. and is not relevant to the choice of reflections for refinement. *R*-factors based on *F*^2^ are statistically about twice as large as those based on *F*, and *R*- factors based on ALL data will be even larger.

### Fractional atomic coordinates and isotropic or equivalent isotropic displacement parameters (Å^2^)

**Table:**

	*x*	*y*	*z*	*U*_iso_*/*U*_eq_	
C1	0.7933 (2)	0.16285 (9)	0.08870 (17)	0.0407 (4)	
C2	0.6198 (2)	0.16921 (10)	0.08652 (19)	0.0466 (4)	
C3	0.5674 (2)	0.10396 (11)	0.1617 (2)	0.0509 (4)	
H3	0.451 (3)	0.1097 (13)	0.161 (3)	0.072 (6)*	
C4	0.6785 (2)	0.03318 (10)	0.2356 (2)	0.0488 (4)	
C5	0.8489 (2)	0.02819 (9)	0.2331 (2)	0.0466 (4)	
H5	0.934 (3)	−0.0186 (12)	0.288 (2)	0.055 (5)*	
C6	0.9094 (2)	0.09227 (9)	0.16142 (18)	0.0421 (4)	
C7	0.4899 (3)	0.24369 (15)	0.0026 (3)	0.0701 (5)	
H7A	0.369 (5)	0.236 (2)	−0.009 (5)	0.139 (12)*	
H7B	0.479 (4)	0.2576 (19)	−0.111 (5)	0.115 (9)*	
H7C	0.530 (4)	0.292 (2)	0.078 (4)	0.111 (9)*	
C8	0.6158 (4)	−0.03631 (15)	0.3157 (3)	0.0718 (6)	
H8A	0.481 (5)	−0.056 (2)	0.237 (5)	0.132 (11)*	
H8B	0.694 (6)	−0.085 (3)	0.348 (5)	0.152 (13)*	
H8C	0.611 (4)	−0.0151 (19)	0.415 (5)	0.116 (9)*	
C9	1.0959 (2)	0.08575 (13)	0.1626 (3)	0.0571 (4)	
H9A	1.083 (3)	0.0893 (13)	0.040 (3)	0.073 (6)*	
H9B	1.161 (4)	0.0329 (16)	0.214 (3)	0.088 (7)*	
H9C	1.179 (3)	0.1299 (15)	0.234 (3)	0.078 (6)*	
N1	0.85323 (19)	0.22763 (8)	0.01026 (18)	0.0489 (4)	
H1	0.868 (3)	0.2161 (12)	−0.078 (3)	0.060 (5)*	
C10	0.9063 (3)	0.30398 (11)	0.0798 (2)	0.0579 (5)	
H10	0.947 (2)	0.3419 (11)	0.007 (2)	0.056 (5)*	
O1	0.9086 (2)	0.33069 (8)	0.21640 (18)	0.0792 (5)	

### Atomic displacement parameters (Å^2^)

**Table:**

	*U*^11^	*U*^22^	*U*^33^	*U*^12^	*U*^13^	*U*^23^
C1	0.0488 (8)	0.0413 (7)	0.0360 (7)	−0.0034 (6)	0.0240 (6)	−0.0044 (5)
C2	0.0491 (8)	0.0497 (8)	0.0436 (8)	0.0045 (6)	0.0247 (6)	−0.0013 (6)
C3	0.0446 (8)	0.0618 (10)	0.0526 (8)	−0.0046 (7)	0.0287 (7)	−0.0060 (7)
C4	0.0568 (9)	0.0480 (8)	0.0452 (8)	−0.0113 (7)	0.0277 (7)	−0.0062 (6)
C5	0.0549 (9)	0.0397 (8)	0.0463 (8)	0.0015 (6)	0.0256 (7)	−0.0010 (6)
C6	0.0443 (7)	0.0450 (8)	0.0387 (7)	−0.0013 (6)	0.0218 (6)	−0.0057 (5)
C7	0.0683 (12)	0.0706 (13)	0.0771 (13)	0.0254 (10)	0.0400 (10)	0.0154 (11)
C8	0.0884 (15)	0.0662 (13)	0.0719 (12)	−0.0216 (11)	0.0480 (12)	0.0007 (10)
C9	0.0499 (9)	0.0671 (11)	0.0600 (10)	0.0033 (8)	0.0313 (8)	−0.0011 (8)
N1	0.0682 (8)	0.0471 (7)	0.0426 (7)	−0.0035 (6)	0.0359 (6)	−0.0011 (5)
C10	0.0854 (12)	0.0489 (9)	0.0507 (8)	−0.0124 (8)	0.0422 (9)	0.0005 (7)
O1	0.1406 (13)	0.0553 (8)	0.0656 (8)	−0.0296 (8)	0.0691 (9)	−0.0155 (6)

### Geometric parameters (Å, °)

**Table:**

C1—C2	1.394 (2)	C7—H7B	0.95 (3)
C1—C6	1.396 (2)	C7—H7C	0.95 (3)
C1—N1	1.4303 (18)	C8—H8A	1.00 (4)
C2—C3	1.385 (2)	C8—H8B	0.95 (4)
C2—C7	1.508 (2)	C8—H8C	0.92 (3)
C3—C4	1.382 (2)	C9—H9A	0.99 (2)
C3—H3	0.94 (2)	C9—H9B	0.97 (3)
C4—C5	1.387 (2)	C9—H9C	0.95 (2)
C4—C8	1.506 (2)	N1—C10	1.325 (2)
C5—C6	1.390 (2)	N1—H1	0.83 (2)
C5—H5	0.96 (2)	C10—O1	1.219 (2)
C6—C9	1.503 (2)	C10—H10	1.023 (18)
C7—H7A	0.94 (4)		
			
C2—C1—C6	121.16 (13)	C2—C7—H7C	113.3 (18)
C2—C1—N1	120.43 (13)	H7A—C7—H7C	100 (3)
C6—C1—N1	118.38 (13)	H7B—C7—H7C	109 (3)
C3—C2—C1	117.98 (14)	C4—C8—H8A	114.8 (19)
C3—C2—C7	120.32 (16)	C4—C8—H8B	114 (2)
C1—C2—C7	121.70 (15)	H8A—C8—H8B	107 (3)
C4—C3—C2	122.68 (14)	C4—C8—H8C	108.2 (19)
C4—C3—H3	120.4 (13)	H8A—C8—H8C	101 (3)
C2—C3—H3	117.0 (13)	H8B—C8—H8C	111 (3)
C3—C4—C5	117.91 (14)	C6—C9—H9A	113.2 (12)
C3—C4—C8	120.91 (16)	C6—C9—H9B	112.6 (14)
C5—C4—C8	121.18 (16)	H9A—C9—H9B	105.7 (19)
C4—C5—C6	121.78 (14)	C6—C9—H9C	110.0 (13)
C4—C5—H5	121.0 (11)	H9A—C9—H9C	107.6 (18)
C6—C5—H5	117.1 (11)	H9B—C9—H9C	107.3 (19)
C5—C6—C1	118.48 (13)	C10—N1—C1	124.38 (12)
C5—C6—C9	120.70 (14)	C10—N1—H1	116.2 (13)
C1—C6—C9	120.83 (14)	C1—N1—H1	119.1 (13)
C2—C7—H7A	113 (2)	O1—C10—N1	126.09 (15)
C2—C7—H7B	110.9 (19)	O1—C10—H10	120.0 (10)
H7A—C7—H7B	111 (3)	N1—C10—H10	113.9 (10)
			
C6—C1—C2—C3	1.1 (2)	C4—C5—C6—C1	−0.7 (2)
N1—C1—C2—C3	178.93 (13)	C4—C5—C6—C9	179.23 (14)
C6—C1—C2—C7	−177.86 (16)	C2—C1—C6—C5	−0.4 (2)
N1—C1—C2—C7	0.0 (2)	N1—C1—C6—C5	−178.22 (12)
C1—C2—C3—C4	−0.9 (2)	C2—C1—C6—C9	179.75 (14)
C7—C2—C3—C4	178.09 (16)	N1—C1—C6—C9	1.9 (2)
C2—C3—C4—C5	−0.1 (2)	C2—C1—N1—C10	70.1 (2)
C2—C3—C4—C8	−179.94 (16)	C6—C1—N1—C10	−112.01 (18)
C3—C4—C5—C6	0.9 (2)	C1—N1—C10—O1	−1.5 (3)
C8—C4—C5—C6	−179.26 (15)		

### Hydrogen-bond geometry (Å, °)

**Table:**

*D*—H···*A*	*D*—H	H···*A*	*D*···*A*	*D*—H···*A*
N1—H1···O1^i^	0.83 (2)	2.05 (2)	2.8775 (18)	171.4 (19)

Symmetry codes: (i) *x*, −*y*+1/2, *z*−1/2.
